# Platelet-activating factor podoplanin: from discovery to drug development

**DOI:** 10.1007/s10555-017-9672-2

**Published:** 2017-07-03

**Authors:** Ai Takemoto, Kenichi Miyata, Naoya Fujita

**Affiliations:** Division of Experimental Chemotherapy, The Cancer Chemotherapy Center, Japanese Foundation for Cancer Research, 3-8-31, Ariake, Koto-ku, Tokyo, 135-8550 Japan

**Keywords:** Podoplanin, Platelet aggregation, Hematogenous metastasis, CLEC-2

## Abstract

Tumor cell-induced platelet aggregation facilitates hematogenous metastasis by promoting tumor embolization, preventing immunological assaults and shear stress, and the platelet-releasing growth factors support tumor growth and invasion. Podoplanin, also known as Aggrus, is a type I transmembrane mucin-like glycoprotein and is expressed on wide range of tumor cells. Podoplanin has a role in platelet aggregation and metastasis formation through the binding to its platelet receptor, C-type lectin-like receptor 2 (CLEC-2). The podoplanin research was originally started from the cloning of highly metastatic NL-17 subclone from mouse colon 26 cancer cell line and from the establishment of 8F11 monoclonal antibody (mAb) that could neutralize NL-17-induced platelet aggregation and hematogenous metastasis. Later on, podoplanin was identified as the antigen of 8F11 mAb, and its ectopic expression brought to cells the platelet-aggregating abilities and hematogenous metastasis phenotypes. From the 8F11 mAb recognition epitopes, podoplanin is found to contain tandemly repeated, highly conserved motifs, designated platelet aggregation-stimulating (PLAG) domains. Series of analyses using the cells expressing the mutants and the established neutralizing anti-podoplanin mAbs uncovered that both PLAG3 and PLAG4 domains are associated with the CLEC-2 binding. The neutralizing mAbs targeting PLAG3 or PLAG4 could suppress podoplanin-induced platelet aggregation and hematogenous metastasis through inhibiting the podoplanin–CLEC-2 binding. Therefore, these domains are certainly functional in podoplanin-mediated metastasis through its platelet-aggregating activity. This review summarizes the platelet functions in metastasis formation, the role of platelet aggregation-inducing factor podoplanin in pathological and physiological situations, and the possibility to develop podoplanin-targeting drugs in the future.

## Platelet aggregation in hematogenous metastasis

Many reports have suggested that platelets are associated with cancer [1, 2]. In cancer patients with advanced disease, venous thromboembolism is frequently occurred. The cancer-associated venous thromboembolism is a serious leading cause of death [3]. Adding to recurrent thromboses increase the risk for cancer, cancer patients are more likely to develop metastasis after thromboembolism experiences. These recent observations more strongly suggest that there is an association between platelet activation and cancer and that activated platelets have a role in cancer progression.

Many agents inhibiting platelet activation, such as the cyclooxygenase inhibitor aspirin, phosphodiesterase inhibitors, and prostacyclin, have been shown to suppress metastasis in experimental animal models [4]. Furthermore, a calcium channel blocker, verapamil, reportedly suppresses platelet aggregation *in vitro* and hematogenous metastasis and spontaneous metastasis in mouse melanoma B16 and mouse colon adenocarcinoma 26 (colon 26) cells without significantly inhibiting the growth of the primary tumors [5]. These evidences suggest that platelet aggregation has a role in hematogenous metastasis. In addition to these findings in experimental models, recent robust clinical analyses have indicated that the anti-platelet agent, aspirin, reduces the frequency of metastasis and increases survival in cancer patients [6, 7]. However, the suppressive effects in clinical studies of other agents—such as the anti-coagulant agent low-molecular-weight heparin—on tumor progression remain controversial. Besides, the experimental metastasis model was developed to evaluate the relationship between tumor-induced platelet aggregation and embolization and metastasis. Tsuruo et al. performed *in vivo* selection in which lungs excised from mice subcatenously (s.c.) transplanted with colon 26 cells were s.c. injected to other mice repeatedly to establish a colon 26-select line (P-select 26) that potentiated the formation of lung metastasis nodules of colon 26 and established subclones from P-select 26 [8]. The characteristics of the subclones derived from P-select 26 were analyzed, and a comparison of subclones with highly metastatic and poorly metastatic potentials showed that platelet aggregation capability was positively correlated with metastasis potential. Mahalingam et al. also isolated subclones of fibrosarcoma, some of which showed high metastatic potential and platelet aggregation ability; however, other subclones showed no correlation between metastasis ability and platelet aggregation ability [9]. Thus, the capacity of a tumor to induce platelet aggregation is among the key factors for hematogenous metastasis formation, although metastasis is not controlled by this capability alone. Lung metastasis in the clones that exhibited high metastasis potentials and platelet aggregation capacity was suppressed by the induction of thrombocytopenia induced by anti-platelet antibodies or neuraminidase or by prostacyclin treatment [9]. These findings suggest that suppressing platelet activation has potential as a treatment for metastasis. However, a more efficient approach is to target the tumor-specific pathway that activates platelets, thereby avoiding the risk for bleeding in patients before and after surgery.

**Fig. 1:**
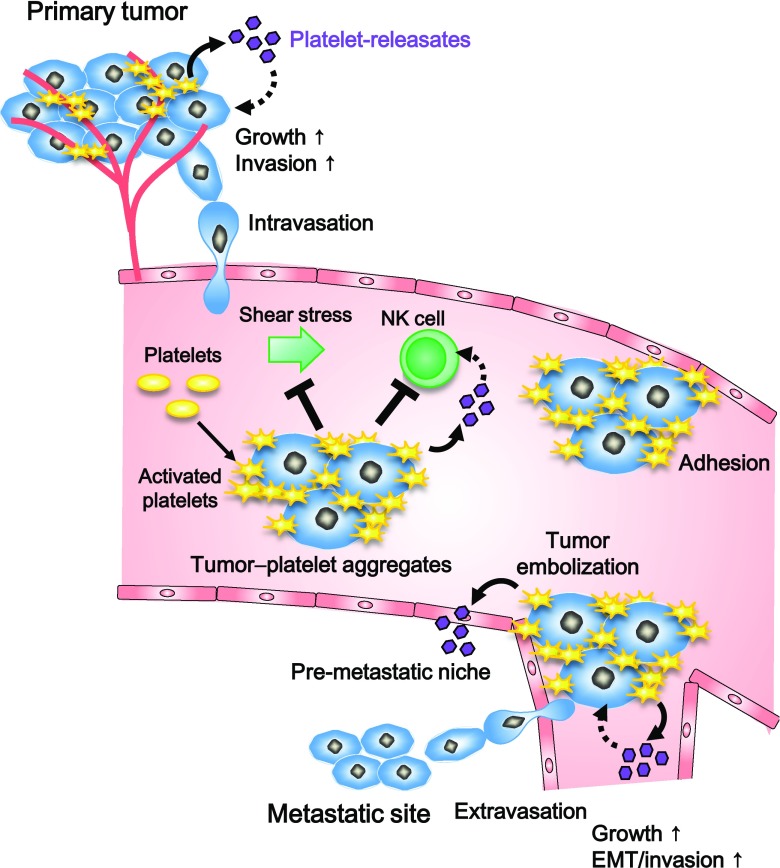
Platelets promote tumor progression through the tumor-induced activation and aggregation. In the circulation, tumor cells interact with platelets and produce tumor–platelet aggregates. The aggregates covered by activated platelets resist against shear stress and suppress immunological assaults by NK cells through the display MHC class I and platelet releasates. Tumor–platelet aggregates prone to adhere and form emboli in microvasculature, which could promote metastasis formation. Platelet releasates also contribute to the formation of pre-metastatic niche and promote tumor growth and metastasis property, EMT/invasion which could contribute to extravasation. Primary tumor possibly interacts with platelets leaked from vessels, and then, activated/aggregated platelets contribute to tumor progression through releasing factors same as in the circulation

So far, many pathways on how platelets promote tumor metastasis are suggested. In hematogenous metastasis, more than 99.9% of intravasated tumor cells die in circulation because they are exposed to shear stress and eliminated by natural killer (NK) cells before reaching the parenchyma of distant tissues [10, 11]. However, some highly metastatic tumor cells can escape these fates by evoking platelet aggregation and building tumor cell–platelet aggregates. The tumor cell–platelet aggregates are easily trapped in microvasculature possibly because of their large size and adhesiveness to vessel wall of activated platelets, which is a prerequisite step for extravasation (Fig. 1). As covered by platelets, tumor cells are prevented from shear stress and immunological elimination (Fig. 1). As reported, the immune surveillance from NK cells is suppressed by some membrane proteins expressed on platelets, such as glucocorticoid-induced tumor necrosis factor receptor-related ligand [12] and MHC class I [13], or by releasing platelet-derived growth factor (PDGF) and transforming growth factor-β (TGF-β) from aggregated platelets [14, 15]. Adding to the effects on immune surveillance, many releasates from activated platelets affect properties of tumor cells and metastatic sites (Fig. 1). The aggregated platelet-derived ATP [16], CXCL5 and CXCL7 [17], and TGF-β [18, 19] support the intravascular extravasation of tumor cells, and aggregated platelet-derived lysophosphatidic acid facilitates the preparation of pre-metastatic niches to promote bone metastasis formation [20]. Not only in the circulation, tumor cells could interact and activate platelets in the primary tumor because of leaky vessels, which suggest that platelet effects come to primary tumor cells. We need to clarify the pathway to tumor progression promoted by platelets and the effect by targeting tumor–platelet pathway on many roles of platelets in pathological but in physiological condition.

## Podoplanin: a novel platelet aggregation-inducing factor

To target tumor-specific platelet activation, identification of factors contributing to tumor–platelet interaction and inducing platelet activation is important. Tumor–platelet interaction depends on several factors: integrin, sialyl Lewis^x^/sialyl Lewis^a^, and so on [1]. Tissue factor expressed by tumor cells can also activate platelets.

To elucidate the factor expressed in tumor cells that induces platelet aggregations, Watanabe et al. immunized rats with the membrane fraction of a highly metastatic subclone (NL-17) derived from the P-select 26 line [8] to establish monoclonal antibody (mAb)-producing hybridomas [21]. A purified mAb designated 8F11 showed higher reactivity toward highly metastatic NL-17 than toward weakly metastatic NL-14, and it inhibited NL-17-induced platelet aggregation. In a mouse melanoma, the mAb showed stronger reactivity to a highly metastatic B16F10 variant than to its original cell line, B16, and it inhibited B16F10-induced platelet aggregation [22]. Furthermore, 8F11 mAb inhibited NL-17-induced experimental lung metastasis [23].

An 8F11 mAb affinity-purified 44 kDa glycoprotein (gp44) induces platelet aggregation *in vitro*, and its platelet aggregation activity is reduced when it is deglycosylated by sequential treatment with neuraminidase and *O*-glycanase [24]. Glycosylation hindered protein identification using mass spectroscopy. Among accumulated annotations of proteins, mouse T1α antigen was expected as a candidate for 8F11 mAb-reactive gp44. The stable expression of mouse T1α antigen on the surface of Chinese hamster ovary (CHO) cells was detected with 8F11 mAb, and the dominant platelet-inducing factor expressed in the NL-17 subclone was identified and designated Aggrus (thereafter, podoplanin) [25]. The expression of mouse podoplanin, a human podoplanin ortholog, also induced platelet aggregation capability in CHO cells.

After the identification of the epitope recognized by the 8F11 mAb and observations of conservation among species, the functional domain critical for platelet aggregation activity was speculated and designated the platelet aggregation-stimulating (PLAG) domain (see Sect. 4). The PLAG is tandemly repeated three times in a conserved manner (PLAG1–3) [26]. Possible *O*-glycosylation sites at Thr34 in PLAG1 and Thr52 in PLAG3 have been suggested to be important for podoplanin-dependent platelet aggregation [25]. Podoplanin is a type I transmembrane sialomucin-like glycoprotein expressed on the cell surface of various tumors and some normal tissues (see Sect. 3). Before its identification as a factor promoting tumor metastasis through platelet aggregation, it had been discovered independently in various mammalian species and given different names: T1alpha as a water channel in humans, mice, and rats [27]; gp40 in dogs [28]; gp36 as a vascular endothelial glycoprotein in humans [29]; OTS-8 as a tumor marker [30]; 8.1.1 mAb antigen [31]; M2A as a D2-40 antigen [32]; and PA2.26 in mice [33] among others. These designations are synonymous with podoplanin.

## Pathological and physiological functions of podoplanin

### Physiological expression and function

For normal condition, podoplanin is expressed on lymphatic endothelial cells, alveolar epithelial type I cells in lung, kidney podocytes, lymph node-derived fibroblastic reticular cells (FRCs), and central nervous system [31, 34–38]. Physiologically podoplanin functions during development, as podoplanin null mice show increased embryonic lethality with disorder in heart development [39] or die after birth owing to respiratory failure and not inflated lung [40, 41]. The defect in the separation of the blood lymphatic vessels is indicated as one of the leading cause [42]. And the C-type lectin-like receptor 2 (CLEC-2) null mice also show the defect in blood lymphatic vessel separation, and the relation of lymphatic podoplanin-mediated platelet aggregation is suggested [43]. In addition, the physiological functions of podoplanin are suggested in postnatal stage. For immune surveillance, lymphocytes enter lymph nodes through specialized blood vessels named high endothelial venules (HEVs). Podoplanin has a role in maintaining HEV barrier function, as the podoplanin deficiency exhibits loss of HEV integrity and spontaneous bleeding in lymph nodes. In this function, the sphingosine-1-phosphate release during podoplanin-mediated platelet aggregation is indicated as a key in maintaining the integrity of HEV [44]. Not only through the platelet interaction, but CLEC-2-expressed dendritic cells cause stretching of stroma by affecting podoplanin-expressed FRC, which leads to lymph node expansion in immune response [45]. And also the interaction between CLEC-2 on megakaryocytes and podoplanin on FRC-like stroma cells is suggested to promote megakaryocyte expansion and proplatelet formation in bone marrow [46]. Recent accumulated reports suggest physiological functions of podoplanin in the development of lymphatic vessels, lymph nodes, and immune responses. We need to know more details about the mechanism for targeting podoplanin in cancer (see Sect. 5).

### Pathological expression and function

Pathologically, enhanced expression of podoplanin in advanced atherosclerotic lesions is suggested to contribute to thrombus formation leading to cardiovascular events [47]. And, podoplanin expression on Th17 cells has a role in the formation of ectopic lymphoid follicles in chronic autoimmune inflammatory diseases [48]. Podoplanin is expressed on various tumor cells, including squamous cell carcinomas (SCCs), glioblastoma, osteosarcoma, bladder carcinoma, mesothelioma, and seminoma [32, 49–53], and its expression correlates with poor prognosis in brain and lung tumors [54–56]. Furthermore, podoplanin expression level correlates with the metastasis in oral SCC and bladder tumors [51, 57]. Not only in tumor cells themselves but also in cancer-associated fibroblasts (CAFs), podoplanin expression is observed and correlated with tumor malignancy and poor prognosis in lung, breast, pancreatic, and liver cancer [58–61]. Podoplanin-expressed CAFs contribute the resistance to epidermal growth factor receptor (EGFR) tyrosine kinase inhibitor gefitinib [62]. Podoplanin-expressed lymph node stromal cells enhance tumor growth *in vivo* by eliminating CD4^+^ tumor-infiltrating lymphocytes that limit the efficiency of tumor immunotherapy [63]. Many reports suggest that podoplanin promotes tumor and metastasis; however, there are several controversial reports that podoplanin expression in lung SCC correlates with lower incidence of lymph node metastasis and good prognosis [64–66].

We described suggested function of activated and aggregated platelets in tumor progression and metastasis in Sect. 1. As described in Sect. 2, podoplanin is a platelet aggregation-stimulating factor; thus, podoplanin expressed in tumor cells could contribute to all pathways induced by platelet aggregation (Fig. 1). In fact, the dependency on podoplanin of tumor embolization [51, 67], and the release of several platelet factors [19, 68, 69], was indicated. The PDGF release by podoplanin-mediated platelet aggregation enhances *in vitro* growth and the resistance for apoptosis through activation of PDGFR-PI3K/Akt pathway in osteosarcoma cells [68]. The TGF-β release by podoplanin-dependent platelet aggregation promotes epithelial–mesenchymal transition (EMT) and invasion of urinary bladder and lung SCC cells [19]. The EGFR ligands containing EGF released by podoplanin-induced platelet aggregation promote lung SCC tumor *in vivo* through the EGFR signal activation [69]. These are reasonable as podoplanin acts in the initial step of those pathways induced by platelet aggregation. Moreover, some platelet-released growth factors, such as TGF-β, basic fibroblast growth factor, and EGF, promote the expression of podoplanin [70]. Thus, the release of these growth factors may further accelerate podoplanin-mediated platelet aggregation and promote tumor growth and metastasis.

## Functional domains and the post-translational modification of podoplanin

### PLAG domains and glycosylation

The PLAG1–3 domains of podoplanin are found as tandemly repeated in a conserved manner, but their contribution in inducing platelet aggregation differs over mammalian species [26]. Possible *O*-glycosylation sites, Thr34 in PLAG1 of mouse podoplanin and Thr34 in PLAG1/Thr52 in PLAG3 of human podoplanin, are implicated in platelet aggregation [25, 26]. Sialyl *O*-glycosylation in podoplanin is central to platelet aggregation-inducing activity, as indicated by studies using enzymatic deglycosylation [24] and podoplanin-expressing CHO mutant series which are deficient in glycosylation pathways [71, 72].

Analyses of the glycosylation site and structure of podoplanin with lectin blot, mass spectrometry, and Edman degradation [71, 72] have revealed that podoplanin has a disialyl-core 1 structure. This sialyl *O*-glycan structure could be introduced into human podoplanin *via* a genetic engineered yeast strain followed by *in vitro* sialylation, and thereafter, the glycosylated podoplanin induced platelet aggregation [73]. A structural analysis of the complex of the sialyl *O*-glycosylated podoplanin PLAG2/3 peptide using the engineered glycosylation system and the extracellular domain of podoplanin counterpart on platelets, CLEC-2, has been reported [74]. From that, Glu47, Asp48, and sialyl-glycosylated Thr52 in the PLAG3 domain were shown to interact with CLEC-2. However, the PLAG3-mutated podoplanin still exhibited platelet aggregation-inducing ability [75]. To answer the discrepancy, another conserved region located at distant with repeated PLAG1–3 domains was identified in human podoplanin as a critical domain for podoplanin-induced platelet aggregation, and designated PLAG4. The analyses using PLAG-mutated podoplanin indicated that PLAG4 domain dominantly contributes human podoplanin-induced platelet aggregation than PLAG3, and the binding of human podoplanin to CLEC-2 depends on PLAG3 and PLAG4 domains [75]. Similarly to PLAG3, the contribution of Glu81, Asp82, and Thr85 of PLAG4 in the CLEC-2 binding was indicated. Thus, “Glu-Asp-(X)-X-X-Thr (ED[X]XXT)” could be the motif required for CLEC-2 binding though sialyl glycosylation in Thr85 of PLAG4 has not been shown.

### Molecular identification of CLEC-2 as a natural podoplanin receptor

CLEC-2 was identified functionally as a platelet receptor for platelet aggregation-inducing snake venom, rhodocytin [76, 77]. Similarities between podoplanin-induced and rhodocytin-induced platelet activation signal pathway in Src kinase and phospholipase Cγ2 (PLCγ2) dependency suggested that they share the same receptor. And analyses showed that podoplanin is an *in vivo* ligand of CLEC-2 [78, 79]. Now, the downstream cascade of podoplanin/CLEC-2 leading to platelet activation is indicated. When podoplanin or rhodocytin binds to CLEC-2, Src family kinase or Syk, or both, phosphorylates tyrosine in the hemi-immunoreceptor tyrosine-based activation motif in the cytoplasmic domain of CLEC-2. Tyrosine phosphorylation is recognized by Syk through its two Src homology 2 (SH2) domains, which results in Syk activation. Activated Syk phosphorylates the LAT or SLP-76 adaptor proteins, which induces the activation of effector enzymes, PLCγ2, and Btk, which leads to platelet aggregation [78, 80].

CLEC-2 expression occurs in platelets, megakaryocytes, neutrophils, monocytes, granulocytes, myeloid, and dendritic cells [81–85]. CLEC-2 null mice reportedly die during the embryonic and neonatal stages with blood-filled lymphatic vessels and edema resulting from defects in blood lymphatic vessel separation [43]. This phenotype is similar to that of podoplanin null mice [43, 86]. However, platelet-specific and megakaryocyte-specific CLEC-2-deficient mice show no embryonic lethality despite having defects in blood lymphatic vessel separation and mild thrombocytopenia [46, 87]. Thus, CLEC-2 expressed in cells other than platelets may play a crucial role in maintaining life at the embryonic and neonatal stages [87]. Podoplanin–CLEC-2 interaction occurs mainly during development and in pathological situations such as tumors. And importantly for targeting podoplanin–CLEC-2 interaction as a therapy, CLEC-2-deficient platelets remain in the activation pathway stimulated by physiological agonists such as thrombin, ADP, and collagen [86]. However, we need to care about indication that podoplanin–CLEC-2 interaction has a crucial role in the moderation of immune response.

## Targeting podoplanin–CLEC-2 interactions for cancer therapy

As described above, the formation of tumor cell–platelet aggregates is central to the process of hematogenous metastasis. The administration of anti-platelet agents to suppress platelet aggregation can increase bleeding risk significantly in cancer patients with thrombocytopenia due to chemotherapeutic drug toxicity. On the other hand, the interaction between podoplanin on tumor cells and CLEC-2 on platelets is hopeful target for suppressing metastasis of podoplanin-positive tumors, because CLEC-2 null platelets show aggregation induced by physiological agonists such as thrombin, ADP, and collagen [86].

**Fig. 2:**
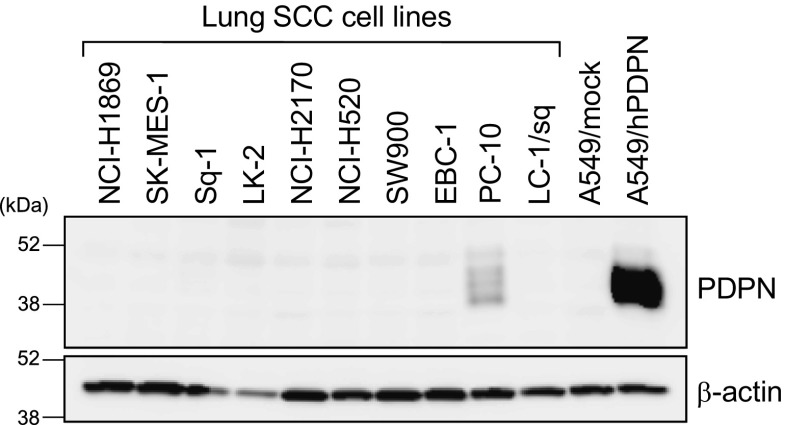
Search for podoplanin-positive lung SCC cell lines. Public available lung SCC cell lines were analyzed by immunoblotting using anti-podoplanin mAb D2-40 (PDPN) and anti-β-actin mAb. Nine out of ten lung SCC cell lines are podoplanin negative except PC-10. This frequency is very low compared with the reported result indicated by immunohistochemistry using clinical samples. The discrepancy of existing cell lines from clinical samples is a critical issue for the evaluation of the tool targeting podoplanin

**Fig. 3:**
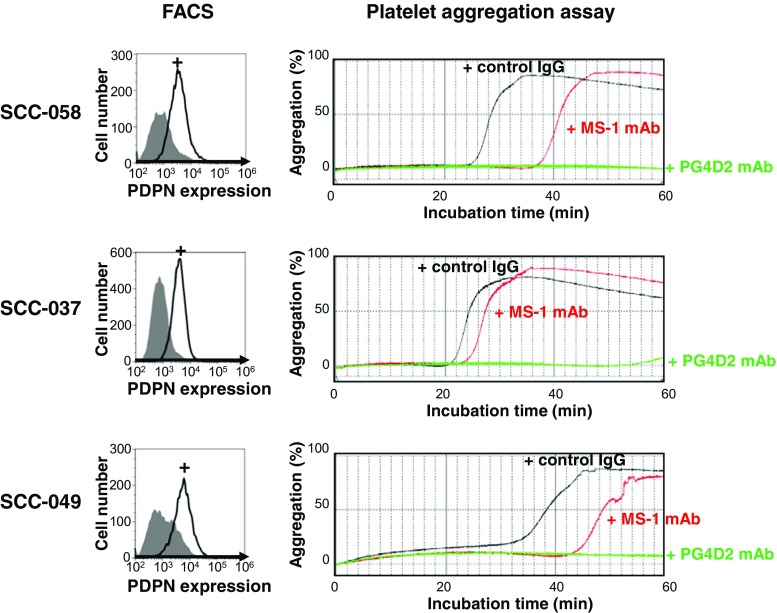
Patient-derived lung SCC cell-induced platelet aggregation depends on podoplanin. Examples of podoplanin-positive lung SCC cells newly established from clinical samples of patients provided informed consent are shown. Their podoplanin (PDPN) expression was analyzed by flow cytometry (FACS) using anti-podoplanin mAb D2-40. *Closed areas* indicate negative control without mAb, and *open areas* indicate D2-40-treated cells; both are treated with anti-mouse IgG-Alexa 488 as a secondary antibody for detection. In the platelet aggregation assays, mouse washed platelets were used. SCC cell-induced platelet aggregation was suppressed by the treatment of cells with anti-podoplanin mAbs (MS-1 and PG4D2) before the addition into assays. Although there are some differences in suppressive effect between MS-1 and PG4D2, and between cells, podoplanin-positive lung SCC cell-induced platelet aggregation depends on podoplanin

Our laboratory and others have developed a variety of anti-podoplanin antibodies to neutralize podoplanin–CLEC-2 interactions and thereby suppress podoplanin-mediated platelet aggregation and hematogenous pulmonary metastasis [75, 88–91]. As human podoplanin uses both PLAG3 and PLAG4 domains for CLEC-2 binding, podoplanin mAbs exhibiting neutralizing ability recognize region in part of PLAG3 or PLAG4 [75]. The monoclonal antibody NZ-1, which recognizes human podoplanin PLAG3, has shown neutralizing activity for podoplanin–CLEC-2 interaction, podoplanin-dependent platelet aggregation, and metastasis [88, 92]. Other established anti-human podoplanin mAbs, P2-0 and MS-1 recognizing the PLAG3 and PG4D2/PG4D2 recognizing PLAG4, suppress platelet aggregation and metastasis by limiting CLEC-2 interaction [75, 90, 91]. Established podoplanin mAbs indicate suppressive effect against platelet aggregation induced by podoplanin-expressed CHO [75] and some podoplanin-positive tumor cells [67, 91]. Moreover, some podoplanin mAbs reportedly show the suppression of *in vivo* hematogenous metastasis not only using podoplanin-expressed CHO cells but also using podoplanin-positive human tumor cell lines [19, 51]. These accumulated evidences indicate that podoplanin mAbs could be developed for clinical use. However, the verification using endogenously podoplanin-expressing tumor cells is still restricted. Available cell lines expressing podoplanin from public cell bank are not many, though podoplanin is expressed on a variety of tumors, which is shown by analyses using clinical samples. And, those cell lines are rarely proper for *in vivo* metastasis model. Actually, when we searched podoplanin-positive lung SCC cell lines which are public available, it turned out that only one cell line PC-10 out of 10 cell lines exhibits podoplanin expression (Fig. 2), even though clinical samples of lung SCCs show more than 60% podoplanin-positive lung SCC cell lines [93]. This suggests that the characteristics of some tumor cells are changed in the process for establishment of cell lines. Recently, we are trying to establish patient-derived cell lines for podoplanin-positive lung SCC and have succeeded to get several lines. The platelet aggregation induced by established podoplanin-positive lung SCC cells is suppressed by anti-podoplanin mAb PG4D2 strongly than by MS-1 (Fig. 3). Analyses using patient-derived model are also required.

**Fig. 4:**
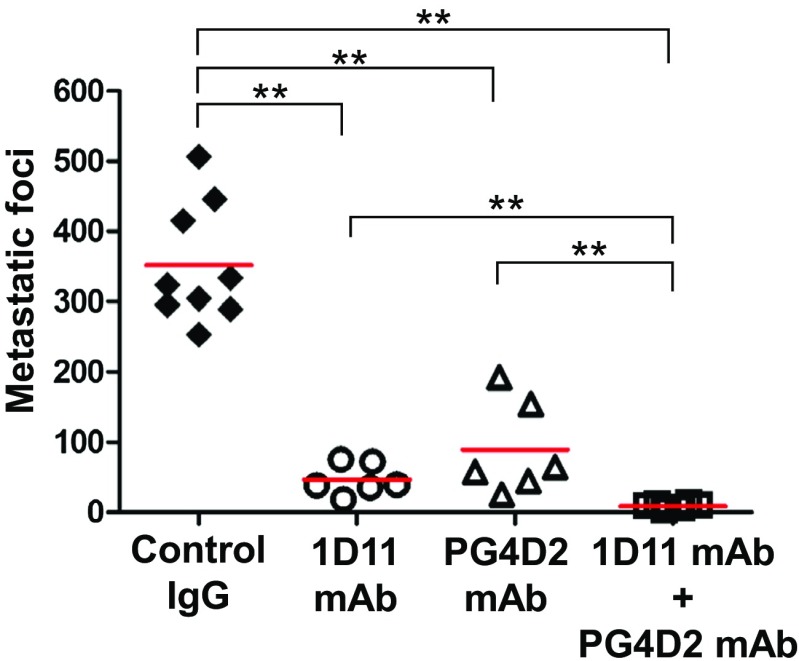
Hematogenous metastasis of urinary bladder SCC cell UM-UC-5 was significantly suppressed by the administration of anti-podoplanin mAb PG4D2 and anti-TGF-β mAb 1D11. Control IgG or PG4D2 mAb (30 μg/mouse) was intravenously (i.v.) injected into tail vein of CB17-SCID mice (♂, 5 weeks) on 1 day before the cell transplantation. Control IgG or 1D11 mAb (100 μg/mouse) was i.v. injected at an hour before the transplantation, and then, UM-UC-5 cells (5 × 10^5^/mouse) was i.v. injected. After 27 days from the cell transplantation, lungs were excised and the metastatic foci on the surface counted after picric acid staining. Mann–Whitney *U* tests were performed. **P < 0.01. Bars represent mean values. Hematogenous metastasis of UM-UC-5 cells depends on podoplanin and TGF-β

As mentioned in Sect. 3, podoplanin-induced platelet aggregation resulted in the release of platelet factors which could promote tumor progression. In fact, the release of PDGF and TGF-β during podoplanin-positive tumor cell-induced platelet aggregation is suppressed by anti-podoplanin mAbs. And the hematogenous metastasis of urinary bladder SCC cell line, UM-UC-5, is suppressed by the administration of anti-podoplanin mAb PG4D and also by that of anti-TGF-β mAb 1D11 (Fig. 4). This might indicate that podoplanin-promoted metastasis pathway depends on TGF-β mainly at least in UM-UC-5. Therefore, searching the downstream pathway of podoplanin leading to metastasis is important for the development of effective strategy for therapy with targeting podoplanin.

Some established anti-podoplanin mAbs, for example, MS-1 and PG4D2, are identified as mouse IgG2a subtype, which exhibits antibody-dependent cellular cytotoxicity/complement-dependent cytotoxicity (ADCC/CDC) activities. At least, MS-1 mAb exhibits anti-tumor activity against podoplanin-positive PC-10 xenograft tumor in immune-deficient NOD-SCID mouse [91], and the recombinant single-chain antibody variable region fragment of MS-1 also suppresses podoplanin-mediated metasiasis [89]. Therefore, the neutralizing ability of anti-podoplanin mAb against podoplanin–CLEC-2 interaction may be enough for suppressing metasiasis or growth of podoplanin-positive tumors. Some anti-podoplanin mAb requires ADCC/CDC activities for suppression of tumor growth and metastasis [94, 95]. We cannot ignore the dependency of ADCC/CDC activities of anti-podoplanin mAbs as podoplanin is expressed in several normal tissues and has some physiological roles (see Sect. 3) and need to care the side effect in developing anti-podoplanin mAb as a therapeutic drug. In that sense, LpMab series, cancer-specific antibodies recognizing cancerous aberrant glycosylated podoplaninan, are potent for therapeutics [96]. Some anti-podoplanin mAbs may be useful as diagnostic tools to identify patients with podoplanin-positive tumor, as higher sensitivity than generally used D2-40, anti-podoplanin mAb [97].

Against many anti-podoplanin mAbs have been established, tools targeting CLEC-2 are only few reported. Targeting CLEC-2 on platelets rather than podoplanin on tumor requires a care for platelet function in hemostasis. A small-molecule compound, 2CP, which is a derivative of 4-*O*-benzoyl-3-methoxy-beta-nitrostyrene, has been reported as a chemical inhibitor of podoplanin-induced platelet aggregation [98]. 2CP exhibits direct binding activity to CLEC-2 and therapeutic efficacy in combination treatment with cisplatin in a mouse metastasis model without causing defects in physiological platelet function in hemostasis. In another recent report, immunological depletion of CLEC-2 by the treatment of mice with anti-CLEC-2 mAb 2A2B10 exhibited suppression of hematogenous metastasis and thrombus formation of podoplanin-positive mouse melanoma cell B16F10 without significant bleeding tendency [99].

More analyses using podoplanin-positive tumor cells and tumor/metastasis model containing the patient-derived model are required to provide insights into the development of new therapies targeting the podoplanin–CLEC-2 interaction.
